# Improve hot region prediction by analyzing different machine learning algorithms

**DOI:** 10.1186/s12859-021-04420-0

**Published:** 2021-10-25

**Authors:** Jing Hu, Longwei Zhou, Bo Li, Xiaolong Zhang, Nansheng Chen

**Affiliations:** 1 School of Computer Science and Technology, Wuhan University of Science and Technology, Wuhan, Hubei China; 2 Hubei Province Key Laboratory of Intelligent Information Processing and Real-Time Industrial System, Wuhan, 430065 Hubei China; 3 Molecular Biology and Biochemistry, Simon Fraser University, Vancouver, BC Canada

**Keywords:** Hot region, Protein–protein interaction, Hot spot, DBSCAN, SVM, Gaussian Naïve Bayes

## Abstract

**Background:**

In the process of designing drugs and proteins, it is crucial to recognize hot regions in protein–protein interactions. Each hot region of protein–protein interaction is composed of at least three hot spots, which play an important role in binding. However, it takes time and labor force to identify hot spots through biological experiments. If predictive models based on machine learning methods can be trained, the drug design process can be effectively accelerated.

**Results:**

The results show that different machine learning algorithms perform similarly, as evaluating using the F-measure. The main differences between these methods are recall and precision. Since the key attribute of hot regions is that they are packed tightly, we used the cluster algorithm to predict hot regions. By combining Gaussian Naïve Bayes and DBSCAN, the F-measure of hot region prediction can reach 0.809.

**Conclusions:**

In this paper, different machine learning models such as Gaussian Naïve Bayes, SVM, Xgboost, Random Forest, and Artificial Neural Network are used to predict hot spots. The experiment results show that the combination of hot spot classification algorithm with higher recall rate and clustering algorithm with higher precision can effectively improve the accuracy of hot region prediction.

**Supplementary Information:**

The online version contains supplementary material available at 10.1186/s12859-021-04420-0.

## Background

Proteins perform their corresponding biological functions by interacting with proteins or other molecules, among which the interactions between proteins are the most important. As the carrier with life activities, protein plays an important role in every link with life, such as gene regulation, signal transduction, gene expression and other basic cellular functions in life activities [1]. The binding between the two proteins is mainly based on affinity. Studies have shown that only a few residues on the protein–protein interaction surface provide the majority of the binding free energy, and these residues are called hot residues [2, 3]. At the same time, these hot residues usually gather closely on the protein interaction surface [4]. That is, hot residues often appear in the form of interaction clusters on the interaction interface, and the residues in these clusters interact with each other to form a stable network structure, which is called the hot region. In drug design, the study of hot region plays a positive role in the prediction of protein functional sites, drug target and protein design [5, 6].

The study showed that the hot region in protein–protein interaction is composed of at least three hot spot residues, which on the protein interaction interface. Starting from the machine learning method, Xia [7] based on protein sequence, structure and neighborhood features to extract feature, combined with maximum relevance and minimum redundancy algorithm, and create a hot spot prediction model based on Support Vector Machine (SVM). Then, Tuncbag [8] proposes an empirical model that combines accessible surface area and pairing propensity to predict hot spots residues, which improves the accuracy of hot spot residue prediction. To achieve better property based on structural features, Huang [9] designed an assembly learning method that combines SMOTE with data imbalance to predict hot spot residues. Hu [10] constructed a new learning hot spot prediction model based on protein sequence feature.

A better hot spot residue prediction model is beneficial to the prediction of hot region. Cukuroglu [11] analyzed hot region according to the characteristics of hot spot residues and the formation rules of hot region, and established a hot region database named Hot Region. Pons uses the small-world residue network to predict hot regions, using the small-world network method, and through the relationship between the residues, the residues can form an interconnected network [12]. Nan [13] used complex network and community detection methods to predict hot region in protein interactions. In the process of predicting hot regions, some False Positives (FP) and False Negatives (FN) in the prediction results are corrected by using the topological characteristics of residues in the network, so as to improve the accuracy of predicting hot regions. An approach based on a new clustering algorithm called Local Community Structure Detecting (LCSD) to identify the hot regions was proposed by Lin [14], with an enhanced maximum relevance minimum redundancy algorithm to upgraded prediction performance in the feature selection process of hot spot prediction.

The prediction of hot spot residues in protein interaction is the first step for predicting hot region. It is necessary to identify the hot spot residue as accurately as possible on the protein–protein interaction. Due to the limitation of amino acid mutation to alanine in the data set and the imbalance between hot spots and non-hot spots in the data set, the prediction effect of hot spots and hot regions in protein–protein interaction is not significant. With the release of the SKEMPI2.0 dataset, there were twice as many mutations to alanine in the SKEMPI2.0 dataset as there were in the previous version of SKEMPI1.0 [15, 16]. From multiple perspectives, we extracted features according to protein sequence, structure and the relationship between amino acid and built several machines learning models to predict hot spots residues. The hot spot residue prediction results of different machine learning algorithms were analyzed, and DBSCAN clustering algorithm was combined to form hot spots [17]. The experiment results show that the combination of hot spot classification algorithm with higher recall rate and clustering algorithm with higher precision can effectively improve the accuracy of hot region prediction.

## Results

### Dataset

The datasets used in this article are from up-to-date SKEMPI 2.0 databases (Structural database of Kinetics and Energetics of Mutant Protein Interactions). The dataset encompasses the variation data of thermodynamic parameters and kinetic rate constant parameters before and after the mutation of amino acids to alanine, leucine and other different types of amino acids. The data in the SKEMPI2.0 dataset are all from experiments or authoritative published literature. Due to different experimental environments, mutations at the same site may have multiple different values of binding free energy in the database, so we use the average value of binding free energy to replace the repeated data and eliminate the empty data. After that, 180 protein complexes were obtained from the SKEMPI2.0 database, and the corresponding structural information of each complex was obtained from the PDB database (Protein Data Bank). Each protein complex consists of a stack of interface residues whose accessible surface area is reduced by more than 1 Å during the formation of the protein complex. We defined hot and non-hot residues according to the energy changes in the alanine mutation experiment of these residues.

In SKEMPI2.0 data set, the average value of ∆∆G are used as the final result for the binding free energy of the same site under different experiments. At present, most of the research on hot spot residues adopts such a definition standard: In the alanine experiment, the interface residues with binding free energy change greater than 2 kcal /mol were regarded as hot spot residues, and the interface residues with binding free energy change less than 0.4 kcal /mol were regarded as non-hot spot residues, and the data that the binding free energy varies between 0.4 kcal/mol and 2.0 kcal/mol are discarded. We found that using 2.0 kcal/mol as the hot spot definition standard of the SKEMPI2.0 data set would cause the data to be extremely unbalanced, resulting in a sharp drop in the recall rate of the hot spot prediction model. The strategy of using 1.0 kcal/mol as the threshold can make better use of the entire data set. Therefore, we define more than 1.0 kcal/mol as hot spot residues, and less than 1.0 kcal/mol as non-hot spot residues.

Using 1.0 kcal/mol as the standard for defining hot and non-hot spots, we finally obtained 2326 interface residues from 180 protein complexes about SKEMPI2.0 database, including 1513 non-hot and 813 hot spot residues. Table 1 shows the specific distribution of 20 amino acids in the data set, which indicates that amino acids with aromatic side chains are more likely to have hot spots residues and TYR, ARG, LYS, and GLU are easier to exist in the hot spot residues. Otherwise, TYR, SER, ARG, and GLU are more likely to appear in the interface residues.

**Table 1:** Distribution of data in SKEMPI 2.0

Amino acid	Non-hot spots	Hot spots	All residues	Ratio of hot spots	property of side chain
SER	143	21	164	0.128	Hydroxyl-containing
CYS	6	1	7	0.143	Sulfur-containing
GLN	107	29	136	0.213	Amid
THR	114	31	144	0.214	Hydroxyl-containing
PRO	42	17	59	0.288	Cyclic
ASN	101	41	142	0.289	Amid
GLY	48	20	68	0.294	Aliphatic
VAL	70	30	100	0.3	Aliphatic
GLU	154	68	222	0.306	Acid
HIS	60	28	88	0.318	Basic aromatic
MET	23	11	34	0.326	Sulfur-containing
LYS	131	64	195	0.328	Basic
ARG	146	80	226	0.354	Basic
ASP	101	41	142	0.409	Acid
LEU	48	41	89	0.419	Aliphatic
ILE	72	52	124	0.461	Aliphatic
PHE	51	55	106	0.519	Aromatic
TYR	75	104	179	0.581	Aromatic
TRP	21	50	71	0.704	Aromatic
All	1513	813	2623	0.35	none

More than two-fifths of the data in the SKEMPI 2.0 database come from the previous version SKEMPI 1.0. Since a lot of research has been done in SKEMPI 1.0, the main work in this article focuses on the dataset extended by SKEMPI 2.0. In order to enhance the stability of the prediction model, in the SKEMPI 2.0 expansion data, we added the protein complex containing the number of interface residues less than 3 into the training set, and put the remaining data of expansion data the test set. The rest of data in SKEMPI 2.0 is regarded as the testing set. Table 2 shows the detailed data of training set and test set.

**Table 2:** Training set and testing set

Dataset	Non-hot spots	Hot spots	All residues	Complexes
Training set	864	390	1254	101
Testing set	649	423	1072	79

### Experimental results

In the hot spot prediction stage, we need to predict the hot spot residues as precisely as possible. The optimal feature subset was obtained through feature selection by mRMR (Max-Relevance and Min-Redundancy) algorithm. The calculated score and details for each feature are in Additional file 1. In this section, we used several traditional machine learning methods to build the hot spot prediction model. such as RF (Random Forest), Xgboost (eXtreme Gradient Boosting Decision Tree), ANN (Artificial Neural Network) and SVM (Support Vector Machine) [18–21], and compared these widely used methods in hot spot research with Gaussian Bayes. For the ANN algorithm, we build a five-layer neural network, in which the activation function of each layer is ReLU (rectified linear units) [22]. Because neural networks have automatic feature fusion functions, in addition to ANN, other machine learning methods all use mRMR feature selection algorithm for feature selection in the feature selection process.

Table 3 shows the prediction results of different machine learning algorithms in the hot spot prediction experiment. According to Table 3, it can be concluded that the accuracy and precision of the Xgboost method is the best compared to other methods. The performance of GNB method on recall and F-measure is the highest. The experimental data showed that GNB could correctly predict more hot spot residues, and Xgboos could correctly predict more non-hot spot residues. Due to hot spot residues are fewer than non-hot spot residues on the training set, the accuracy of GNB is lower than that of Xgboost. The more hot spots residues are predicted, the more hot regions we can get during clustering process.

**Table 3:** Comparison of results with different methods to predict hot spots

Methods	Hot Spot			
	Accuracy	Recall	Precision	F-measure
GNB	0.674	**0.792**	0.561	**0.657**
SVM	0.696	0.6	0.618	0.609
Xgboost	**0.726**	0.596	**0.672**	0.632
RF	0.693	0.596	0.615	0.605
ANN	0.715	0.679	0.632	0.655

The highest value in each column is shown in bold

**Fig. 1:**
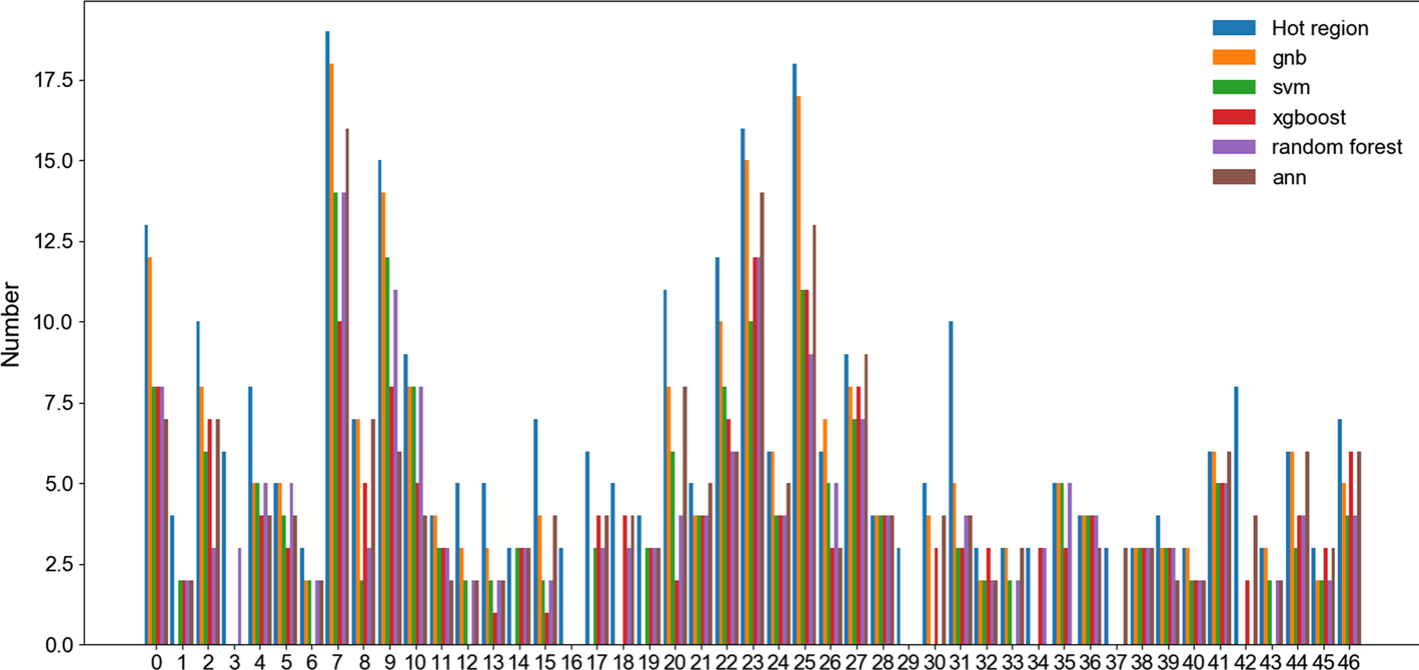
Distribution of hot spots in hot regions. The x-axis corresponds to the 47 standard hot regions in Additional file 2. The height of the bar shows the number of hot spots in those 47 standard hot regions and the number of true positive hot spots in the predicted hot regions

After the hot spot residue prediction, DBSCAN clustering algorithm was used to predict the hot region. The clustering results are presented in Table 4. The F-measure represents the balance between recall and precision, using F-measure as the evaluation criterion, the two parameters “Min” and “ε” in the DBSCAN algorithm are determined by the grid search method. Experiment results show that the hot region prediction model combined with GNB and DBSCAN algorithms is significantly better than other methods. The Fig. 1 showed that the number of hot spot residues correctly predicted by GNB in a single hot region of 47 standard hot regions is close to the performance of other algorithms. The more true positive hot spot residues are predicted, the more hot region will be correctly constructed. Otherwise, few hot spots will cause the recall rate of hot region prediction to decrease. Besides, because of the lack of true positive residues, some hot regions are incorrectly clustered in the process of forming true positive hot regions with relatively unconstrained parameters.

**Table 4:** Comparison of results with different methods to predict hot regions

Methods	Hot Region		
	Recall	Precision	F-measure
GNB	**0.766**	**0.923**	**0.809**
SVM	0.617	0.725	0.667
Xgboost	0.574	0.6	0.587
RF	0.617	0.644	0.63
ANN	0.596	0.538	0.567

The highest value in each column is shown in bold

### Standard hot regions and predicted hot regions

In this paper, we define the standard hot region according to Keskin [23]. The definition of a standard hot region: Each hot region is composed of at least three hot spot residues, each hot spot residue is assumed to be a regular sphere, and the Cα atom of each hot spot residue is considered to be the center of the sphere. Calculate the radius of the sphere from the volume of the hot spot residue sphere [24]. If the distance between the centers of two spheres (two Cα-atoms of two hot spots) is less than the sum of the radius of the two spheres plus a tolerance distance (2 Å), the two hot spot residues are flagged to be clustered and to form a network in the hot region. The prediction accuracy of the prediction model for different amino acid mutations is compared with the standard hot regions. Finally, 47 standard hot regions were detected. For detailed results, see Additional file 2.

Because of a hot region contains at least three hot spot residues, and the maximum distance between two contacting amino acids in the same hot region is 9.5 Å, in the process of setting the parameters of "Min" and "ε" of DBSCAN algorithm, we set "Min" to be greater than 3 and set "ε" to be less than 9.5 Å. According to the results of all methods clustering, when "Min" is 3 and "ε" is 9.5 Å, the DBSCAN algorithm could achieves the optimal F-measures in other methods except the GNB. However, under this parameter setting, GNB recognizes more non-hot spots as hot spots. With "Min" set to 4 and "ε" set to 8.5 Å, the GNB could more accurately label non-hot spot as noises. The clustering algorithm will perform well when the factors that form hot regions are improved.

## Methods

The hot spot residue that was predicted by different machine learning algorithm are clustered to form hot region with DBSCAN algorithm. Then, we evaluate the hot region prediction by comparing hot regions from predicted models with the standard hot regions in dataset.

### Binding free energy changes

Data on single amino acids mutated into alanine was extracted from the SKEMPI 2.0 (https://life.bsc.es/pid/skempi2/) which contains affinities of wild type complexes and affinities of mutated complexes measured by biological experiments in the scientific literature. Because binding free energy changes of multiple mutated residues has not been accumulated based on such single mutated residues, more samples from multiple mutated samples cannot be deduced. The data with affinity could not be measured were deleted. We calculate the bind free energy of amino acid mutations according to Formula (1), where the binding affinity (${\text{K}}_{d}$) is determined according to biological experiments such as Surface Plasmon Resonance and Isothermal Titration Calorimetry [25, 26], and R is the gas constant (8.314/4184) kcal/(K*mol) (1 kcal = 4.184 kJ) and T is the experimental temperature (in the range of 273 K to 323 K). The change of bind free energy ($\Delta \Delta G$) can be calculated based on the $\Delta {\text{G}}_{mut}$ and $\Delta {\text{G}}_{wt}$, which be calculated from Eqs. (1), (2).

1 $$\Delta {\text{G}} = - {\text{RT}}\ln ({\text{Kd}})$$

2 $$\Delta \Delta G = \Delta G_{mut} - \Delta G_{wt}$$

### Feature selection

In this paper, the structural information on protein complexes is from PDB (Protein Data Bank) [27]. We extracted features including solvent accessible surface area, protrusion index, relative accessible surface area, binding sites and the depth index with aimed amino acid from PSAIA [28], and calculate RctASA, RctmPI by formula (3), (4), conservation scores from the ConSurf server [29], the attributes of amino acid side chains, the hydrophobic index, and the interaction numbers between two amino acids. The detailed features are shown in Additional file 1. The additional file indicated that the attribution of the amino acid side chain is a discrete variable, we encode the feature with one-hot, which is a feature extraction method that can deal with discontinuous numerical features. In total, we collected 83 features from protein structural, sequence and energy. Since not every feature contributes the same to the model, we determined the optimal feature subset by combining mRMR algorithm (minimum Redundancy Maximum Relevance) [30]; the mutual information I (x, y) is labeled as:

3 $$RctASA = \frac{{\left[ {unbound\,total\,ASA} \right] - \left[ {bound\,total\,ASA} \right]}}{{\left[ {unbound\,total\,ASA} \right]}}$$

4 $$RctmPI = \frac{{\left[ {unbound\,total\,mean\,PI} \right] - \left[ {bound\,total\,mean\,PI} \right]}}{{\left[ {unbound\,total\,mean\,PI} \right]}}$$

5 $$I\left( {x,y} \right) = \iint {P\left( {x,y} \right)\log \frac{{P\left( {x,y} \right)}}{p\left( x \right)p\left( y \right)}dxdy}$$

and the maximum correlation criterion and the minimum redundancy criterion are defined as:

6 $$\max D\left( {F,c} \right),D = \frac{1}{F}\mathop \sum \limits_{Xi \in F} I\left( {Xi,c} \right)$$

7 $$\min R\left( F \right),R = \frac{1}{{F^{2} }}\mathop \sum \limits_{Xi,Xj \in F} I\left( {Xi,Xj} \right)$$

According to the training set, a feature list is produced. To discover the highest F-score combination, we applied incremental feature selection and got a rank of all features in descending order. Every time we combined the feature ranked at the top with its next one to obtain the F-measure in machine learning models, then selected the set of features with the best F-measure.

### Prediction of hot regions

There are non-hot spot residues and hot spot residues in the dataset. However, we needed to detect as many hot spots as possible in the hot regions which contain hot spot residues.

### Naïve Bayes classifier

We constructed a Gaussian Naïve Bayes classifier given a set of training examples with class labels and then used the model to distinguish between non-hot spot residues and hot spot residues [31–33]. One example is a tuple of features $\left( {x_{1} ,x_{2} , \ldots ,x_{n} } \right)$ of one sample $\left( {x_{i} } \right)$ and the class label c of the sample, so X is all samples and C is the classification variable. In our experiment, we assumed that there are two classes: c = 0 (non-hot spot residue) and c = 1 (hot spot residue). According to the Bayes Rule, the probability of one sample ${\text{E}} = \left( {x_{1} ,x_{2} , \ldots ,x_{n} } \right)$, being class c of sample E is:

8 $$p(c|E) = \frac{p(E|c)p\left( c \right)}{{p\left( E \right)}}$$

Sample E is classified as class 1 if and only if $f_{b} \left( E \right)$ is more than 1, otherwise, sample E will be classified as class 0:

9 $$f_{b} \left( E \right) = \frac{p(C = 1|E)}{{p(C = 0|E)}} \ge 1$$

where $f_{b} \left( E \right)$ is called a Bayesian classifier.

Assuming that all features are independent given the value of the class variable, that is

10 $$p(E|c) = p(x_{1} ,x_{2} , \ldots ,x_{n} |c) = \mathop \prod \limits_{i = 1}^{n} p(x_{i} |c)$$

the resulting classifier is then:

11 $$f_{nb} \left( E \right) = \frac{{p\left( {C = 1} \right)}}{{p\left( {C = 0} \right)}}\mathop \prod \limits_{i = 1}^{n} \frac{{p(x_{i} |C = 1)}}{{p(x_{i} |C = 0)}}$$

The function $f_{nb} \left( E \right)$ is called a Naïve Bayesian classifier, or simply Naïve Bayes (NB). When we used the Gaussian distribution to calculate the $p(x_{i} |C)$, the classifier becomes Gaussian Naïve Bayes. Given probability distribution is under Gaussian distribution, the function is:

12 $$g\left( {x_{i} ,\mu ,\sigma } \right) = \frac{1}{{\sqrt {2\pi \sigma } }}e^{{ - \frac{{\left( {x_{i} - \mu } \right)}}{{2\sigma^{2} }}}}$$

### Support vector machine

A support vector machine constructs a hyper-plane or set of hyper-planes in a high or infinite-dimensional space, which can be used for classification, regression or other tasks. Intuitively, a good separation is achieved by the hyper-plane that has the largest distance to the nearest training data points of any class (so-called functional margin), since in general the larger the margin the lower the generalization error of the classifier. Given training dataset $D = \left\{ {\left( {x_{1} ,y_{1} } \right), \ldots ,\left( {x_{n} ,y_{n} } \right)} \right\}$ the goal of the classification is to find a maximum-margin hyperplane $w^{t} x + b = 0$.

Used as the output of SVM in binary classification:

13 $$f\left( {x,W} \right) = {\text{sgn}} \left( {\mathop \sum \limits_{i = 1}^{N} w_{i} K(x_{j} ,x_{i} ) + b} \right)$$

optimized objective function:

14 $$\begin{aligned} & J = W^{T} W = \left\| W \right\|^{2} \\ & {\text{s.t.:}}\,\,y_{J} \left[ {\mathop \sum \limits_{i = 1}^{N} w_{i} K\left( {x_{j} ,x_{i} } \right) + b} \right] \ge 1, j = 1, \ldots ,N \\ \end{aligned}$$

where N is sample size, W is the output adjustable parameter vector of support vector machine, $K\left( {x_{j} ,x_{i} } \right)$ is kernel function.

The objective function J is to ensure the optimality of classification, and the constraint condition is to ensure the correctness of classification. In order to eliminate the influence of noise and abnormal samples, relaxation variables are introduced as follows:

15 $${\text{J}} = \frac{1}{2}W^{T} W + C\mathop \sum \limits_{i = 1}^{N} \xi_{j}$$

16 $$y_{j} \left[ {\mathop \sum \limits_{i = 1}^{N} w_{i} K\left( {x_{j} ,x_{i} } \right) + b} \right] \ge 1 - \xi_{j} , j = 1, \ldots N,\xi_{j} \ge 0$$

### Xgboost

Xgboost (eXtreme Gradient Boosting) is one type of ensemble learning. The boosting method, by combining multiple weak learners, gives the final learning result. We used the theory of regression tasking to build the optimal Boosting model, regardless of the classification or regression.

17 $${\text{Obj}} = \mathop \sum \limits_{i = 1}^{n} l\left( {y_{i} ,\hat{y}_{i} } \right) + \mathop \sum \limits_{k = 1}^{K} \Omega \left( {f_{k} } \right)$$

The objective function consists of two parts, the first part is used to measure the difference between the predicted score and the real score, and the second part is the regularization term.

The newly generated tree is to fit the residual error predicted last time, that is, when t trees are generated, the prediction score can be written as:

18 $$\hat{y}_{i}^{\left( t \right)} = \hat{y}_{i}^{{\left( {t - 1} \right)}} + f_{t} \left( {\gamma_{i} } \right)$$

where t is the number of leaf nodes, and w is the fraction of leaf nodes. Gamma can control the number of leaf nodes; a lambda can control the number of leaf nodes so they do not get too large as this avoids overfitting.

According to (17) (18) the objective function:

19 $${\text{Obj}}^{\left( t \right)} = \mathop \sum \limits_{i = 1}^{n} l\left( {y_{i} ,\hat{y}_{i}^{{\left( {t - 1} \right)}} + f_{t} \left( {X_{i} } \right)} \right) + \Omega \left( {f_{t} } \right)$$

in Xgboost is used to approximate it using its Taylor second-order expansion at $f_{t} = 0$. Therefore, the objective function is approximate:

20 $${\text{Obj}}^{\left( t \right)} \simeq \mathop \sum \limits_{i = 1}^{n} \left[ {l\left( {y_{i} ,\hat{y}_{i}^{{\left( {t - 1} \right)}} } \right) + g_{i} f_{t} \left( {X_{i} } \right) + \frac{1}{2}h_{i} f_{t}^{2} \left( {x_{i} } \right)} \right] + \Omega \left( {f_{t} } \right)$$

where $g_{i}$ is a derivative, and $h_{i}$ is the second derivative.

The objective function can be simplified as:

21 $$\widetilde{{{\text{Obj}}}}^{\left( t \right)} \simeq \mathop \sum \limits_{i = 1}^{n} \left[ {g_{i} f_{t} \left( {X_{i} } \right) + \frac{1}{2}h_{i} f_{t}^{2} \left( {x_{i} } \right)} \right] + \Omega \left( {f_{t} } \right)$$

### Random forest

The advantage of a random forest algorithm is that it combines several weak classifiers into one strong classifier, which can resist the over-fitting of the decision tree by using a voting mechanism. Random forest has a strong generalization ability and high efficiency and accuracy for multidimensional data classification. The Gini coefficient is defined as follows:

22 $${\text{Gini}}\left( p \right) = \mathop \sum \limits_{k = 1}^{K} p_{k} \left( {1 - p_{k} } \right) = 1 - \mathop \sum \limits_{k = 1}^{K} p_{k}^{2}$$

where $p_{k}$ is the probability that the sample is in class k. The probability of misclassification is (1-*p*_*k*_). The Gini was calculated for each feature in each sample, and then we selected the feature with the optimal Gini coefficient, theta $\theta^{*}$.

23 $$\theta^{*} = \min Gini\left( {\theta_{i} } \right)$$

where $\theta_{i} { }$ represents the feature of the i in the sample.

### Artificial neural network

An artificial neural network (ANN) is a computational model based on the structure and functions of biological neural networks. Information that flows through the network affects the structure of the ANN because a neural network changes or learns, based on the input and output. We constructed networks containing five layers with activation function rectified linear units (ReLu) and the input layer size corresponds to the 83 features obtained from feature selection.

### Evaluation

In this paper, we adopt the following general evaluation indicators to evaluate the performance of the prediction model for hot spots and hot region.

24 $${\text{Recall}} = {\text{TP}}/\left( {{\text{TP}} + {\text{FN}}} \right)$$

25 $${\text{Precision}} = {\text{TP}}/\left( {{\text{TP}} + {\text{FP}}} \right)$$

26 $${\text{F-measure}} = 2*{\text{Recall}}*{\mathrm{Precision}}/\left( {{\text{Recall}} + {\text{Precision}}} \right)$$

When predicting hot spots, the following notations are used:

- True Positive (TP): The number of hot spots in predicted hot regions and also in standard hot regions;
- False Negative (FN): The number of hot spots that are not in predicted hot regions but in standard hot regions;
- False Positive (FP): The number of hot spots in predicted hot regions but not in standard hot regions;
- Precision represents the accuracy of the hot spot prediction, and Recall represents the coverage of predicted hot spots in standard hot regions. With a good balance between Precision and Recall, the F-measure offers a better overall accuracy of hot spot prediction.

However, for prediction of hot regions, the following notations are used:

- True Positive (TP): The number of hot regions in predicted hot regions and also in standard hot regions;
- False Negative (FN): The number of hot regions that are not in predicted hot regions but in standard hot regions;
- False Positive (FP): The number of hot regions in predicted hot regions but not in standard hot regions;

Similarly, Precision represents the accuracy of the hot region prediction, and Recall represents the coverage of predicted hot regions in standard hot regions. With a good balance between Precision and Recall, the F-measure offers a better overall accuracy in predicting hot regions than using either Precision or Recall solely.

### Cluster

Cluster analysis is abbreviated as clustering, which is the process of dividing a data object into subsets. Each subset is a cluster. The clustering process makes the objects in the clusters similar to each other, but not similar to the objects in other clusters. Because the hot spot residues in protein–protein interactions are not evenly distributed on the interface of protein interactions, they are tightly gathered in a dense area. We use the DBSCAN (Density-Based Spatial Clustering of Applications with Noise) algorithm to predict hot spots based on the characteristic that hot spot residues gather on the protein interaction interface in a flowing structure. The DBSCAN algorithm is suitable to cluster this kind of data. There are two hyperparameters “Min” and “ε” to be measured in this algorithm. “Min” represents the density of residue measured by the number of residues of it, and “ε” represents the radius of residue O as the center of a circle.

For a dataset D composed of residues with the parameters of “Min” and “ε”, the residues with more than or equal to “Min” will be regarded as the core residue in its ε-neighborhood. After checking all residues, the core residues and their ε-neighborhood residues will make up the dense regions, which are the clusters we need.

The detailed process about clustering is that all residues should be defined as “unvisited” in the first stage. Then, we need to select randomly one residue p as the center of the circle and calculate the number of residues in its neighborhood to distinguish whether the residue is a core residue or not. If it is a core residue, we labelled the residue as “visited” and selected the neighborhood residues as the next detected objects. If existing core residues are in them, the process continues until the cluster C cannot be extended. Then we return to the beginning, select randomly one residue p in the remaining residues that was labeled as “unvisited” as the center of a circle, and repeat the process until all residues are “visited”. Eventually, we will obtain several clusters.
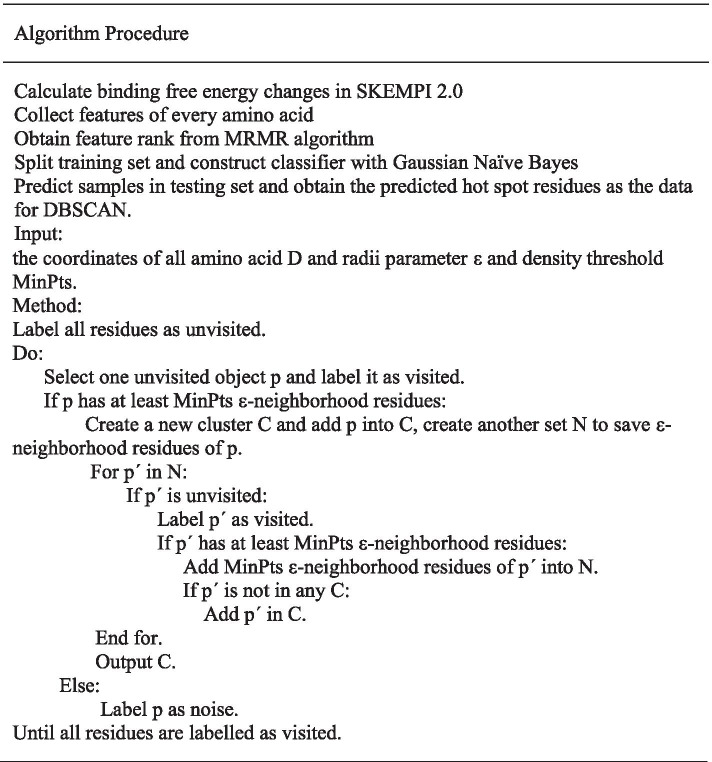


## Discussion

### Comparison of prediction results visualization

**Fig. 2:**
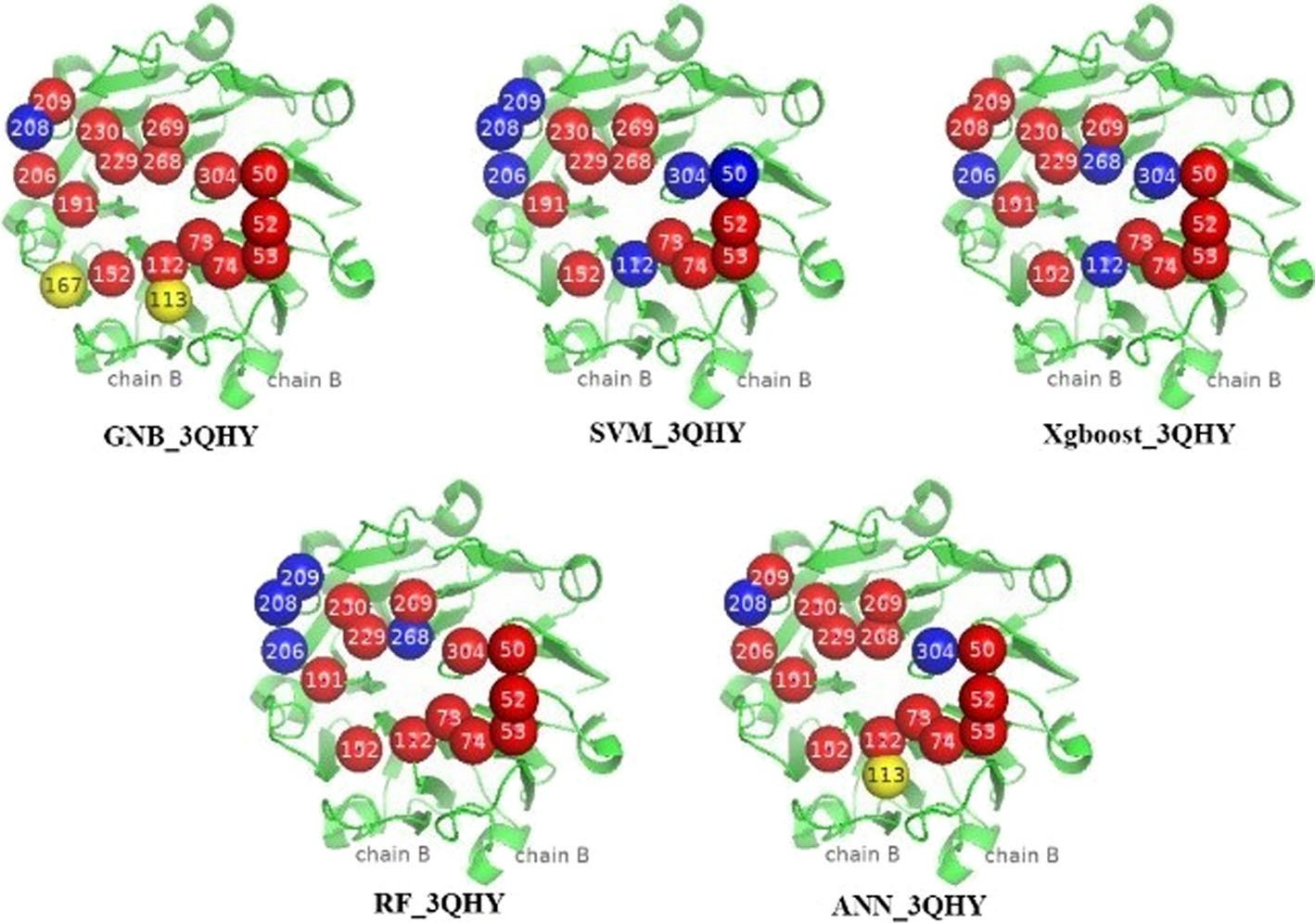
Visual prediction results with PyMol of 3HQY using different methods. All residues are in chain B. Red spheres are predicted correctly in the standard hot region, Blue spheres are not predicted in the standard hot region, yellow spheres are mistaken as hot spot residues

We assumed that a hot region is predicted correctly only when 60 percent of hot spot residues in the standard hot regions occur in the predicted hot region. Therefore, if the results predicted by GNB are clustered to form a hot region that are regarded as true positive hot regions, the evaluation of the results of other machine learning methods for recall will be reduced, and the increase of mistaken hot regions will lead to the evaluation of precision is decrease. We visualized the hot spot residues and predicted hot spot residues of the protein complex 3HQY with PyMol software [34] in Fig. 2. In the protein complex 3QHY, there are 16 hot spot residues in the standard hot region. The GNB algorithm can correctly predict 15 true positive hot spot residues in the standard hot region and only two non-hot spot residues come within the predicted hot region. In addition to ANN, other models have higher accuracy for non-hot spot residues, but cannot predict more hot spot residues in the standard hot region, so the recall is low. ANN can correctly predict 14 hot spot residues in the standard hot region, but a non-hot spot residue was incorrectly predicted as a hot spot residue.

## Conclusion

In this paper, we collect alanine mutations data from the latest presented SKEMPI 2.0 database. When we use 1.0 kcal/mol as the threshold for hot spot and non-hot spot residues, it shows that amino acids of aromatic are more likely to become hot spots residues. Furthermore, hot spot residues are 70.4% from TRP. In the first stage, we used the mRMR algorithm to rank the importance of every feature based on mutual information and RctmPI is the most important feature. In the next stage of predicting hot spot residues, the performance of all methods about F-measure is close, but Gaussian Naïve Bayes (GNB) has the best performance for recall, so that hot regions can be made up of enough true positive hot spot residues. In the final stage, the DBSCAN algorithm was selected to cluster the data for forming hot regions.

The combined method with Gaussian Naïve Bayes (GNB) and DBSCAN can effectively improve hot region predictions. Though several machine learnings methods are applied to test the performance, the limitation of the method is barely biological experiments involved. Thus, the next step is to collect and apply more biological data to verify the model.

## Supplementary Information

**Additional file 1:** Result of MRMR algorithm rank.

**Additional file 2:** Standard hot regions and detailed experimental results for hot spot and hot region prediction.

## Data Availability

The SKEMPI2.0 database is available at (https://life.bsc.es/pid/skempi2/). In the research, part of the core code and data are available at (https://github.com/nsiakjdw/Paper-datasets.git). The datasets used and analyzed during the current study are available from the corresponding author on reasonable request.

## Abbreviations

PPIs: Protein–Protein Interactions
PDB: Protein Data Bank
GNB: Gaussian Naïve Bayes
SVM: Support Vector Machine
Xgboost: EXtreme Gradient Boosting Decision Tree
RF: Random Forest
ANN: Artificial Neural Network
