# Supplementary material for: Improve hot region prediction by analyzing different machine learning algorithms
Source: BMC Bioinformatics. 2021 Oct 25;22(Suppl 3):522. doi: 10.1186/s12859-021-04420-0 (PMC8543831; doi:10.1186/s12859-021-04420-0)
Supplement: Supplementary file 1 — Additional file 1: Result of MRMR algorithm rank. [file 12859_2021_4420_MOESM1_ESM.docx]

Appendix 1. MRMR Algorithm rank

| mRMR features  Order Fea Name Score |
| --- |
| 1 9 RctmPI 0.173  2 68 one_hot8 247.221  3 42 polar_RASA_unbound 8.084  4 3 residue_id 2.608  5 64 one_hot4 3.380  6 80 LEU 3.721  7 77 GLY 2.938  8 88 TYR 3.047  9 87 TRP 3.137  10 60 conservation 3.121  11 83 PHE 3.174  12 33 minimum_CX_bound 3.324  13 89 VAL 3.004  14 81 LYS 3.104  15 73 ASP 3.213  16 24 side_chain_mean_DPX_bound 3.360  17 71 ARG 3.278  18 78 HIS 3.457  19 72 ASN 3.295  20 66 one_hot6 3.339  21 79 ILE 3.392  22 25 side_chain_standard_deviation_DPX_bound 3.284  23 86 THR 3.214  24 75 GLU 2.903  25 76 GLN 2.989  26 82 MET 2.994  27 84 PRO 2.974  28 85 SER 2.739  29 63 one_hot3 2.598  30 10 RcsmPI 2.648  31 57 binding_count 2.383  32 31 side_chain_standard_deviation_CX_bound 2.199  33 67 one_hot7 2.046  34 59 different 2.023  35 17 total_RASA_bound 2.010  36 8 RcsASA 1.760  37 58 same 1.631  38 30 side_chain_mean_CX_bound 1.502  39 23 total_standard_deviation_DPX_bound 1.345  40 7 RctASA 1.351  41 69 one_hot9 1.326  42 74 CYS 1.337  43 15 polar_ASA_bound 1.287  44 38 no_polar_ASA_unbound 1.147  45 70 ALA 1.124  46 11 RcpASA 1.138  47 20 polar_RASA_bound 1.094  48 22 total_mean_DPX_bound 1.054  49 28 total_mean_CX_bound 1.027  50 21 no_polar_RASA_bound 0.930  51 27 minimum_DPX_bound 0.901  52 29 total_standard_deviation_CX_bound 0.865  53 56 hydrophobility_unbound 0.841  54 19 side_chain_RASA_bound 0.819  55 13 back_bone_ASA_bound 0.792  56 32 maximum_CX_bound 0.760  57 55 minimum_CX_unbound 0.734  58 16 no_polar_ASA_bound 0.733  59 26 maximum_DPX_bound 0.671  60 51 total_standard_deviation_CX_unbound 0.673  61 12 total_ASA_bound 0.566  62 18 back_bone_RASA_bound 0.536  63 52 side_chain_mean_CX_unbound 0.533  64 14 side_chain_ASA_bound 0.511  65 62 one_hot2 0.470  66 53 side_chain_standard_deviation_CX_unbound 0.425  67 61 one_hot1 0.404  68 36 side_chain_ASA_unbound 0.381  69 46 side_chain_mean_DPX_unbound 0.315  70 9 minimum_DPX_unbound 0.291  71 45 total_standard_deviation_DPX_unbound 0.289  72 65 one_hot5 0.285  73 37 polar_ASA_unbound 0.269  74 54 maximum_CX_unbound 0.244  75 41 side_chain_RASA_unbound 0.223  76 35 back_bone_ASA_unbound 0.216  77 34 total_ASA_unbound 0.199  78 48 maximum_DPX_unbound 0.182  79 43 no_polar_RASA_unbound 0.164  80 47 side_chain_standard_deviation_DPX_unbound 0.140  81 40 back_bone_RASA_unbound 0.136  82 50 total_mean_CX_unbound 0.131  83 39 total_RASA_unbound 0.099 |
