# Supplementary material for: Improve hot region prediction by analyzing different machine learning algorithms
Source: BMC Bioinformatics. 2021 Oct 25;22(Suppl 3):522. doi: 10.1186/s12859-021-04420-0 (PMC8543831; doi:10.1186/s12859-021-04420-0)
Supplement: Supplementary file 2 — Additional file 2: Standard hot regions and detailed experimental results for hot spot and hot region prediction. [file 12859_2021_4420_MOESM2_ESM.docx]

Appendix 2. standard hot regions in testing data

|  | PDB | Hot Regions |
| --- | --- | --- |
| 0 | 1A07 | A65ARG, A66LYS, A70HIS, A74HIS, A159TYR, A166GLU, A167TRP, C5TYR, C8TYR, D98THR, D101TRP, E30GLU, E103PRO |
| 1 | 1BD2 | C5TYR, C8TYR, D94GLU, E30GLU |
| 2 | 1BP3 | A18HIS, A21HIS, A25PHE, A58ILE, A167ARG, A168LYS, A172LYS, A174GLU, A176PHE, A178ARG |
| 3 | 1C1Y | B59ARG, B65LYS, B66GLN, B67ARG, B68THR, B69VAL |
| 4 | 1C4Z | A635LEU, A645TYR, A653MET, D62PRO, D63PHE, D96LYS, D100LYS |
| 5 | 1E50 | A106MET, A109ASN, A149THR, A161THR, B104ASN |
| 6 | 1GUA | A37GLU, A39SER, B59ARG |
| 7 | 1JTD | A102LEU, A105TYR, A107PRO, A111LYS, A129MET, B50ASN, B52ASP, B53TRP, B73TYR, B74PHE, B112ASN, B131ASP, B152TRP, B191TYR, B229ILE, B230PHE, B268GLU, B269TRP, B304ASN |
| 8 | 1K8R | A38ASP, A41ARG, A57ASP, B81GLN, B83ARG, B101LYS, B160ARG |
| 9 | 1MI5 | D(30A)ASP, D33HIS, D48HIS, D50LEU, D93PRO, D94LEU, D97GLY, D100TYR, D102GLY, D103LYS, E31SER, E97GLY, E98GLN, E100TYR, E106GLN |
| 10 | 2AK4 | A157ARG, C4PRO, C5LEU, C6PRO, C7GLN, D50ASN, D93SER, D96TYR, D97ASN |
| 11 | 2BNR | A65LYS, A66LYS, A70HIS, A73THR, |
| 12 | 2NYY | A917PHE, A918ASN, A919LEU, A1056LYS, A1058ASP |
| 13 | 2NZ9 | A917PHE, A918ASN, A919LEU, A1056LYS, A1058ASP |
| 14 | 3BV4 | C57LEU, C60PHE, C61LEU |
| 15 | 3C60 | C40LYS, C66VAL, D4GLN, D5LYS, D7LYS, D103THR, D107HIS |
| 16 | 3D3V | C8TYR, E30GLU, E98LEU |
| 17 | 3EQS | B2SER, B3PHE, B5GLU, B6TYR, B7TRP, B10LEU |
| 18 | 3EQY | C3PHE, C5GLU, C6TYR, C7TRP, C10LEU |
| 19 | 3LB6 | A14ILE, A104LYS, A105LYS, A107PHE |
| 20 | 3M62 | A40ASP, A44LEU, A49GLU, A96GLY, A97TYR, A104ILE, B45ILE, B47SER, B50VAL, B52GLN, B69VAL |
| 21 | 3M63 | A40ASP, A97TYR, A100VAL, A104ILE, A107PHE |
| 22 | 3NGB | H47TRP, H50TRP, H55GLY, H57VAL, H58ASN, H71ARG, H73VAL, H100TYR, H(100A)ASN, H(100B)TRP, L91TYR, L96GLU |
| 23 | 3QHY | B50ASN, B52ASP, B53TRP, B73TYR, B74PHE, B112ASN, B152TRP, B191TYR, B206ASP, B208TYR, B209PHE, B229ILE, B230PHE, B268GLU, B269TRP, B304ASN |
| 24 | 3QIB | B64GLN, B66GLU, B69GLU, P7TYR, P9LYS, P12YHR |
| 25 | 3S9D | A15LEU, A26LEU, A30LEU, A33ARG, A144ARG, A148MET, A149ARG, A152SER, A153LEU, B43TYR, B44THR, B45ILE, B46MET, B77GLU, B80VAL, B100TRP, B103ILE, B104ASP |
| 26 | 3SE4 | B152LYS, C44THR, C46MET, C77GLU, C80VAL, C103ILE |
| 27 | 3SE8 | H47TRP, H50TRP, H52LYS, H57VAL, H58SER, H61ARG, H62GLN, H(100D)PHE, L96GLU |
| 28 | 3SE9 | H47TRP, H50TRP, H57ASN, H64ARG |
| 29 | 3SEK | C57LEU, C60PHE, C61LEU |
| 30 | 3SZK | C125TYR, C126TYR, C127HIS, C128PHE, C129PHE |
| 31 | 4G0N | A35THR, A36ILE, A37GLU, A38ASP, B59ARG, B65LYS, B66GLN, B67ARG, B68THR, B69VAL |
| 32 | 4HFK | B63LEU, B64GLU, D91THR |
| 33 | 4HRN | D25LEU, D52VAL, D55PHE |
| 34 | 4I77 | H98TYR, H100TYR, L91ASN |
| 35 | 4JFF | C4GLY, C6GLY, C7ILE, C8LEU, C9THR |
| 36 | 4OFY(1) | A54GLN, A60PHE, A105GLN, D61LEU |
| 37 | 4OFY(2) | D53GLN, D105GLN, D115ARG, |
| 38 | 4OZG | H108PHE, H109ARG, H114ASP |
| 39 | 4P23(1) | C39LYS, C61GLN, C62ASN, C65VAL |
| 40 | 4P23(2) | D-19LYS, D-21LYS, D70ARG |
| 41 | 4P5T | C60LEU, C62ASN, D-19LYS, D-21LYS, D-22GLN, D-24GLU |
| 42 | 4PWX | A246PHE, A250TYR, A257GLN, A258GLU, B322ARG, C30TYR, C37ARG, C42ARG |
| 43 | 4RS1 | A109ASN, A112ASP, A115LEU |
| 44 | 5C6T | A280TYR, A290PHE, A292GLU, H102ASN, H103TYR, H104PHE |
| 45 | 5M2O | A79LEU, B43VAL, B47GLN |
| 46 | 5XCO | B6PRO, B7LEU, B8TYR, B9ILE, B10SER, B11TYR, B12ASP |

Prediction hot regions in different methods:

SVM

| PDB | Hot Regions |
| --- | --- |
| 1AO7 | A63GLU, A66LYS, A67VAL, A70HIS, A73THR, A152VAL, A155GLN, A159TYR, A163THR, A167TRP, C1LEU, C5TYR, C8TYR, D28GLY, D31SER, D93THR, D99ASP, D100SER, D101TRP, E95ARG, E101GLY, E103PRO |
| 1BD2 | C5TYR, D94GLU, E104TYR |
| 1BP3 | A21HIS, A164TYR, A167ARG, A168LYS, A171ASP, A172LYS, A176PHE, A178ARG, A179ILE |
| 1C4Z | A635LEU, A638SER, A639LEU, A642LEU, A653MET, A655ILE, A656THR, A660SER, A690PHE, D6ARG, D62PRO, D63PHE, D97PRO |
| 1E50 | A66ASP, A69ASN, A106MET, A113TYR, A114SER, A149THR, A159VAL, A161THR, B63ASN, B65SER, B104ASN |
| 1GUA | A37GLU, A39SER, B68THR, B69VAL |
| 1JTD | A102LEU, A105TYR, A107PRO, A110GLU, A111LYS, A216VAL, B52ASP, B53TRP, B73TYR, B74PHE, B152TRP, B191TYR, B229ILE, B230PHE, B268GLU, B269TRP |
| 1K8R | A37GLU, A38ASP, A39SER, B83ARG |
| 1MI5 | D30THR, D31TYR, D33HIS, D48HIS, D50LEU, D94LEU, D96GLY, D97GLY, D100TYR, D102GLY, E31SER, E97GLY, E98GLN, E100TYR, E106GLN |
| 2AK4 | A69THR, C4PRO, C5LEU, C6PRO, C7GLN, D29THR, D31TYR, D50ASN, D93SER, D96TYR, D97ASN, E30ASN, E98LEU |
| 2BNR | A63GLU, A66LYS, A67VAL, A68LYS, A70HIS, A72GLN, A73THR, A76VAL |
| 2NYY | A918ASN, A919LEU, A920GLU, A953PHE, A954ASN, A1063THR, A1064HIS |
| 2NZ9 | A918ASN, A919LEU, A920GLU, A953PHE, A954ASN, A1062ASP, A1063THR, A1064HIS |
| 3B4V | C57LEU, C60PHE, C61LEU |
| 3C60 | C62GLN, C63ASN, C66VAL, D5LYS |
| 3EQS | B3PHE, B7TRP, B10LEU |
| 3LB6 | A14ILE, A104LYS, A107PHE |
| 3M62 | A44LEU, A96GLY, A97TYR, B45ILE, B47SER, B69VAL, B71MET |
| 3M63 | A44LEU, A96GLY, A97TYR, A100VAL, A104ILE, A107PHE |
| 3NGB | H47TRP, H50TRP, H54GLY, H55GLY, H57VAL, H58ASN, H100BTRP, L91TYR, L96GLU |
| 3QHY | B52ASP, B53TRP, B73TYR, B74PHE, B152TRP, B191TYR, B229ILE, B230PHE, B268GLU, B269TRP |
| 3QIB | B69GLU, B77THR, B81HIS, P7TYR, P9LYS, P12THR |
| 3S9D | A15LEU, A26LEU, A33ARG, A148MET, A149ARG, A152SER, B44THR, B46MET, B47SER, B80VAL, B100TRP, B103ILE |
| 3SE3 | A131LEU, A132ASP, A135SER, A181THR, A238PHE, A241ARG |
| 3SE4(1) | A70TYR, A131LEU, A132ASP, A135SER, A181THR, A238PHE, A241ARG |
| 3SE4(2) | B28PRO, B28PRO, B148MET, B148MET, B152LYS, B152LYS, C44THR, C46MET, C80VAL |
| 3SE8 | H30ARG, H47TRP, H50TRP, H53LEU, H54TRP, H55GLY, H57VAL, H58SER, H61ARG, H100CASP, H100DPHE, L91PHE, L96GLU, L97PHE |
| 3SE9 | H34LEU, H47TRP, H50TRP, H53THR, H54GLY, H56VAL, H57ASN, H59GLY, H64ARG, H99THR, H100GLY, H100AGLY, H100CGLY, L91LEU, L96GLU |
| 4G0N | A21ILE, A37GLU, A39SER, B68THR, B69VAL, B88VAL |
| 4HFK | B40ARG, B63LEU, B64GLU, B66SER, B74GLU |
| 4HRN | D25LEU, D51SER, D52VAL |
| 4JFF | C1GLU, C4GLY, C6GLY, C7ILE, C8LEU, C9THR |
| 4OFY | A54GLN, A56MET, A60PHE, A105GLN, A107SER, A108GLU, D61LEU |
| 4OZG | H57GLN, H108PHE, H109ARG, H110PHE, H114ASP |
| 4P23 | C61GLN, C62ASN, C65VAL, D-17ASN, D-19LYS, D-21LYS |
| 4P5T | C61GLN, C62ASN, C65VAL, C72VAL, D-17ASN, D-19LYS, D-21LYS, D-22GLN, D-24GLU, D77THR |
| 4RS1 | A48ASP, A99GLN, A112ASP, A115LEU |
| 5C6T | A280TYR, A283THR, A297PHE, A299ILE, H102ASN, H104PHE, L50TYR |
| 5M2O | A75ASN, A77LYS, A121HIS, B39ILE, B40SER, B43VAL, B47GLN, B83GLN, B87LEU |
| 5XCO | B6PRO, B7LEU, B9ILE, B12ASP |

Xgboost

| PDB | Hot Regions |
| --- | --- |
| 1AO7 | A66LYS, A70HIS, A167TRP, C1LEU, C5TYR, C8TYR, D50TYR, D99ASP, D100SER, D101TRP, E30GLU, E95ARG, E101GLY, E103PRO |
| 1BD2 | C5TYR, D94GLU, E104TYR |
| 1BP3 | A21HIS, A164TYR, A167ARG, A168LYS, A171ASP, A172LYS, A174GLU, A176PHE, A178ARG, A179ILE |
| 1C4Z | A635LEU, A638SER, A639LEU, A653MET, A655ILE, A659ILE, A690PHE, D6ARG, D60GLU, D62PRO, D63PHE, D97PRO |
| 1E50 | A106MET, A113TYR, A159VAL, A161THR, B63ASN, B104ASN |
| 1JTD(1) | A102LEU, A104GLU, A105TYR, A107PRO, A110GLU, B73TYR, B152TRP, B191TYR, B229ILE, B230PHE, B268GLU, B269TRP |
| 1JTD(2) | A216VAL, B50ASN, B53TRP |
| 1K8R | A37GLU, A38ASP, A39SER, A41ARG, B81GLN, B83ARG, B101LYS |
| 1MI5 | D31TYR, D48HIS, D50LEU, D94LEU, D97GLY, D100TYR, E31SER, E50GLN, E98GLN, E100TYR |
| 2AK4 | A154GLU, A155GLN, C4PRO, C5LEU, C7GLN, C10LEU, D96TYR, D97ASN, E30ASN, E98LEU |
| 2B0U | C47PHE, C51ILE, C52PHE |
| 2BNR | A65ARG, A66LYS, A68LYS, A70HIS, A72GLN |
| 2NYY | A954ASN, A1062ASP, A1063THR, A1064HIS |
| 2NZ9 | A919LEU, A920GLU, A1062ASP, A1063THR, A1064HIS |
| 3B4V | C57LEU, C60PHE, C61LEU |
| 3C60 | C62GLN, C63ASN, C66VAL, D10LYS |
| 3EQS | B3PHE, B6TYR, B7TRP, B10LEU |
| 3EQY | C3PHE, C6TYR, C7TRP, C10LEU |
| 3HH2 | C47PHE, C51ILE, C52PHE |
| 3LB6 | A14ILE, A104LYS, A107PHE |
| 3M62 | B9PHE, B45ILE, B50VAL, B71MET |
| 3M63 | A44LEU, A97TYR, A100VAL, A104ILE, A107PHE |
| 3NGB | H47TRP, H50TRP, H57VAL, H58ASN, H61ARG, H100BTRP, L91TYR, L96GLU, L97PHE |
| 3QHY | B50ASN, B52ASP, B53TRP, B73TYR, B74PHE, B152TRP, B191TYR, B208TYR, B209PHE, B229ILE, B230PHE, B269TRP |
| 3QIB | B66GLU, B76ASP, B77THR, B81HIS, P7TYR, P9LYS, P12THR |
| 3S9D | A26LEU, A30LEU, A33ARG, A144ARG, A148MET, A149ARG, B44THR, B46MET, B47SER, B77GLU, B80VAL, B100TRP |
| 3SE3 | A70TYR, A131LEU, A238PHE, A241ARG |
| 3SE4(1) | A70TYR, A131LEU, A132ASP, A181THR, A238PHE, A241ARG |
| 3SE4(2) | B32LEU, B148MET, C44THR, C46MET, C80VAL |
| 3SE8 | H30ARG, H47TRP, H50TRP, H52LYS, H53LEU, H54TRP, H57VAL, H61ARG, H62GLN, H64GLN, H71ARG, H100CASP, H100DPHE, L91PHE, L96GLU, L97PHE |
| 3SE9 | H47TRP, H50TRP, H53THR, H57ASN, H61PRO, H64ARG, H71ARG, H73ARG, H98TYR, H100GLY, H100AGLY, L91LEU, L96GLU |
| 3SZK | C125TYR, C126TYR, C129PHE |
| 4G0N | A21ILE, A36ILE, A38ASP, B67ARG, B88VAL |
| 4HFK | B40ARG, B63LEU, B64GLU, B74GLU, D91THR |
| 4I77 | H97TYR, H98TYR, H100TYR, L91ASN |
| 4JFF | C6GLY, C7ILE, C8LEU |
| 4OFY | A54GLN, A60PHE, A105GLN, A108GLU, D61LEU |
| 4OZG | H108PHE, H109ARG, H110PHE, H114ASP |
| 4P23 | C55ASP, C61GLN, C62ASN, C65VAL, C68HIS, D-16LYS, D-19LYS, D-21LYS |
| 4P5T | C57GLN, C61GLN, C62ASN, C65VAL, D-16LYS, D-19LYS, D-21LYS, D-22GLN, D-24GLU |
| 4PWX | B69LYS, B322ARG, C30TYR |
| 5C6T | A280TYR, H102ASN, H103TYR, H104PHE |
| 5F4E | A160ARG, B45GLU, B62TRP |
| 5M2O | A75ASN, A77LYS, A79LEU, A121HIS, B39ILE, B40SER, B43VAL, B47GLN, B83GLN, B87LEU |
| 5XCO | B6PRO, B7LEU, B8TYR, B9ILE, B11TYR, B12ASP |

RF

| PDB | Hot Regions |
| --- | --- |
| 1AO7 | A63GLU, A66LYS, A67VAL, A70HIS, A73THR, A152VAL, A155GLN, A159TYR, A163THR, A167TRP, C1LEU, C5TYR, C8TYR, D28GLY, D31SER, D93THR, D99ASP, D100SER, D101TRP, E95ARG, E101GLY, E103PRO |
| 1BD2 | C5TYR, D94GLU, E104TYR |
| 1BP3 | A21HIS, A164TYR, A171ASP, A176PHE, A178ARG, A179ILE |
| 1C1Y | B66GLN, B68THR, B69VAL |
| 1C4Z | A635LEU, A638SER, A639LEU, A642LEU, A653MET, A655ILE, A656THR, A660SER, A690PHE, D6ARG, D62PRO, D63PHE, D97PRO |
| 1E50 | A69ASN, A106MET, A109ASN, A113TYR, A114SER, A149THR, A159VAL, A161THR, B63ASN, B65SER, B104ASN |
| 1GUA | A37GLU, A39SER, B66GLN, B68THR, B69VAL |
| 1JTD | A99GLN, A102LEU, A105TYR, A107PRO, A110GLU, A216VAL, B50ASN, B52ASP, B73TYR, B112ASN, B152TRP, B191TYR, B229ILE, B230PHE, B268GLU, B269TRP, B304ASN |
| 1K8R | A38ASP, A39SER, B81GLN, B83ARG |
| 1MI5 | D30THR, D31TYR, D48HIS, D50LEU, D94LEU, D96GLY, D97GLY, D100TYR, D102GLY, E31SER, E50GLN, E97GLY, E98GLN, E100TYR, E106GLN |
| 2AK4 | A155GLN, C4PRO, C5LEU, C6PRO, C7GLN, D31TYR, D50ASN, D93SER, D96TYR, D97ASN, E30ASN, E98LEU |
| 2B0U | C47PHE, C51ILE, C52PHE |
| 2BNR | A63GLU, A66LYS, A67VAL, A68LYS, A70HIS, A72GLN, A73THR, A76VAL |
| 2NYY | A918ASN, A919LEU, A920GLU, A953PHE, A954ASN, A1063THR, A1064HIS |
| 2NZ9 | A918ASN, A919LEU, A920GLU, A953PHE, A954ASN, A1062ASP, A1063THR, A1064HIS |
| 3B4V | C57LEU, C60PHE, C61LEU |
| 3C60 | C62GLN, C63ASN, C66VAL, D5LYS, D9ASN |
| 3EQS | B3PHE, B7TRP, B10LEU |
| 3EQY | C3PHE, C7TRP, C10LEU |
| 3LB6 | A14ILE, A104LYS, A107PHE |
| 3M62 | A96GLY, A97TYR, B45ILE, B47SER, B71MET |
| 3M63 | A44LEU, A96GLY, A97TYR, A100VAL, A104ILE, A107PHE |
| 3NGB | H47TRP, H50TRP, H58ASN, H61ARG, H100BTRP, L91TYR, L96GLU |
| 3QHY | B50ASN, B52ASP, B53TRP, B73TYR, B74PHE, B112ASN, B152TRP, B191TYR, B229ILE, B230PHE, B269TRP, B304ASN |
| 3QIB | B64GLN, B77THR, B81HIS, P7TYR, P9LYS, P12THR |
| 3S9D | A26LEU, A33ARG, A148MET, A152SER, B44THR, B46MET, B47SER, B76HIS, B80VAL, B100TRP, B103ILE |
| 3SE3 | A70TYR, A131LEU, A132ASP, A135SER, A181THR, A238PHE, A241ARG |
| 3SE4(1) | A70TYR, A131LEU, A132ASP, A181THR, A238PHE, A241ARG |
| 3SE4(2) | B28PRO, B28PRO, B148MET, B148MET, B152LYS, B152LYS, C44THR, C46MET, C80VAL |
| 3SE8 | H30ARG, H47TRP, H50TRP, H54TRP, H55GLY, H57VAL, H58SER, H61ARG, H100CASP, H100DPHE, L91PHE, L96GLU, L97PHE |
| 3SE9 | H34LEU, H47TRP, H50TRP, H53THR, H54GLY, H56VAL, H57ASN, H64ARG, H99THR, H100GLY, H100AGLY, H100CGLY, L91LEU, L96GLU |
| 4G0N | A21ILE, A37GLU, A39SER, B66GLN, B68THR, B69VAL, B88VAL |
| 4HFK | B40ARG, B63LEU, B64GLU, B66SER |
| 4HRN | D25LEU, D51SER, D52VAL |
| 4I77 | H98TYR, H100TYR, L91ASN |
| 4JFF | C1GLU, C4GLY, C6GLY, C7ILE, C8LEU, C9THR |
| 4OFY | A54GLN, A56MET, A60PHE, A105GLN, A107SER, A108GLU, D61LEU |
| 4OZG | H57GLN, H108PHE, H109ARG, H110PHE, H114ASP |
| 4P23 | C61GLN, C62ASN, C65VAL, D-17ASN, D-19LYS, D-21LYS |
| 4P5T | C61GLN, C62ASN, C65VAL, D-17ASN, D-19LYS, D-21LYS, D-22GLN, D-24GLU |
| 4RS1 | A48ASP, A99GLN, A112ASP, A115LEU |
| 5C6T | A280TYR, A297PHE, A299ILE, H102ASN, H103TYR, H104PHE, L50TYR |
| 5F4E | A160ARG, B45GLU, B62TRP |
| 5M2O | A68ASN, A75ASN, A77LYS, A121HIS, A124ASN, B39ILE, B40SER, B43VAL, B47GLN, B83GLN, B87LEU |
| 5XCO | B6PRO, B7LEU, B9ILE, B12ASP |

ANN

| PDB | Hot Regions |
| --- | --- |
| 1AO7 | A58GLU, A65ARG, A66LYS, A152VAL, A154GLU, A155GLN, A159TYR, A166GLU, A167TRP, C5TYR, D99ASP, D101TRP, E95ARG, E101GLY, E102ARG |
| 1BD2 | C5TYR, D94GLU, E104TYR |
| 1BP3 | A21HIS, A160TYR, A164TYR, A167ARG, A168LYS, A171ASP, A172LYS, A174GLU, A176PHE, A178ARG, A179ILE, A180VAL |
| 1C4Z | A634VAL, A635LEU, A638SER, A641ASP, A653MET, A655ILE, D33LEU, D60GLU, D62PRO, D63PHE, D64LYS, D97PRO |
| 1E50 | A66ASP, A69ASN, A106MET, A113TYR, A114SER, A149THR, A159VAL, A161THR, B63ASN, B104ASN |
| 1GUA | A37GLU, A39SER, B66GLN |
| 1JTD | A102LEU, A104GLU, A105TYR, A107PRO, A110GLU, A111LYS, A216VAL, B50ASN, B52ASP, B53TRP, B73TYR, B74PHE, B112ASN, B113TYR, B152TRP, B191TYR, B229ILE, B230PHE, B268GLU, B269TRP |
| 1K8R | A37GLU, A38ASP, A39SER, A41ARG, A57ASP, B81GLN, B83ARG, B101LYS, B160ARG |
| 1MI5 | D31TYR, D48HIS, D50LEU, D94LEU, D100TYR, E50GLN, E98GLN, E100TYR |
| 2AK4 | A154GLU, A155GLN, C5LEU, C6PRO, C7GLN, D31TYR, D96TYR, E30ASN |
| 2B0U | C47PHE, C51ILE, C52PHE |
| 2BNR | A65ARG, A66LYS, A68LYS, A72GLN |
| 2NYY | A918ASN, A919LEU, A920GLU, A953PHE, A954ASN, A1062ASP, A1064HIS |
| 2NZ9 | A918ASN, A919LEU, A920GLU, A953PHE, A1062ASP, A1063THR, A1064HIS, A1294ARG |
| 3B4V | C57LEU, C60PHE, C61LEU |
| 3C60 | C58GLN, C61LEU, C62GLN, C66VAL, D4GLN, D5LYS, D7LYS, D10LYS, D96ARG |
| 3EQS | B3PHE, B6TYR, B7TRP, B10LEU |
| 3EQY | C3PHE, C6TYR, C7TRP, C10LEU |
| 3F1S | A71MET, A74ASP, A238ASP, A239LYS, A240TYR |
| 3HH2 | C47PHE, C51ILE, C52PHE |
| 3LB6 | A14ILE, A104LYS, A107PHE |
| 3M62 | A40ASP, A44LEU, A96GLY, A97TYR, B9PHE, B45ILE, B47SER, B50VAL, B69VAL |
| 3M63 | A40ASP, A44LEU, A97TYR, A100VAL, A104ILE, A107PHE |
| 3MZG | A27HIS, A30HIS, A173HIS, A180HIS |
| 3NGB | H47TRP, H50TRP, H57VAL, H58ASN, H59TYR, H61ARG, H100BTRP, L96GLU, L97PHE |
| 3QHY | B50ASN, B52ASP, B53TRP, B73TYR, B74PHE, B112ASN, B113TYR, B152TRP, B191TYR, B206ASP, B209PHE, B229ILE, B230PHE, B268GLU, B269TRP |
| 3QIB | B66GLU, B69GLU, B76ASP, B77THR, B81HIS, P7TYR, P9LYS, P12THR |
| 3S9D | A15LEU, A26LEU, A27PHE, A30LEU, A33ARG, A144ARG, A148MET, A149ARG, A152SER, B46MET, B47SER, B76HIS, B77GLU, B80VAL, B100TRP, B103ILE |
| 3SE3 | A70TYR, A131LEU, A132ASP, A238PHE, A241ARG |
| 3SE4(1) | A70TYR, A131LEU, A132ASP, A238PHE, A241ARG |
| 3SE4(2) | B28PRO, B32LEU, B148MET, B152LYS, C46MET, C47SER, C80VAL |
| 3SE8(1) | H30ARG, H47TRP, H50TRP, H52LYS, H53LEU, H54TRP, H55GLY, H57VAL, H58SER, H61ARG, H62GLN, H64GLN, H71ARG, H100CASP, H100DPHE, L50ASP, L91PHE, L96GLU, L97PHE |
| 3SE8(2) | H75GLN, H76APRO, H76DPRO |
| 3SE9 | H47TRP, H50TRP, H52BVAL, H53THR, H54GLY, H56VAL, H57ASN, H59GLY, H64ARG, H71ARG, H73ARG, H74ASP, H98TYR, H100GLY, H100AGLY, H100CGLY, L3VAL, L31GLY, L91LEU, L96GLU, L97PHE |
| 3SZK | C125TYR, C126TYR, C127HIS, C129PHE |
| 4B0M | M43LEU, M103PRO, M104PRO |
| 4G0N | A21ILE, A37GLU, A38ASP, A39SER, B66GLN, B67ARG, B84LYS |
| 4HFK | B40ARG, B63LEU, B64GLU, B74GLU |
| 4HRN | D25LEU, D52VAL, D55PHE |
| 4L3E | A155GLN, A166GLU, D50TYR |
| 4OFY(1) | A54GLN, A56MET, A60PHE, A108GLU, D61LEU |
| 4OFY(2) | D53GLN, D105GLN, D115ARG |
| 4OZG | H57GLN, H108PHE, H109ARG, H110PHE, H114ASP |
| 4P23 | C55ASP, C57GLN, C60LEU, C61GLN, C65VAL, D-16LYS, D-19LYS, D-21LYS, D-24GLU |
| 4P5T | C55ASP, C57GLN, C60LEU, C61GLN, C62ASN, C65VAL, D-16LYS, D-19LYS, D-21LYS, D-22GLN, D-24GLU |
| 4PWX | A246PHE, A250TYR, B69LYS, B322ARG, C30TYR, C31GLU |
| 4RS1 | A48ASP, A99GLN, A112ASP, A115LEU, B195LYS |
| 5C6T | A280TYR, A290PHE, A292GLU, A297PHE, A299ILE, H32SER, H102ASN, H103TYR, H104PHE, L50TYR |
| 5E6P | B66ILE, B68LEU, B88ARG |
| 5F4E | A160ARG, B45GLU, B62TRP |
| 5M2O | A75ASN, A77LYS, A79LEU, A121HIS, A124ASN, B39ILE, B40SER, B43VAL, B47GLN, B83GLN, B87LEU |
| 5XCO | B6PRO, B7LEU, B8TYR, B9ILE, B11TYR, B12ASP |

GNB

| PDB | Hot Regions |
| --- | --- |
| 1AO7 | A63GLU, A65ARG, A66LYS, A67VAL, A70HIS, A73THR, A74HIS, A152VAL, A155GLN, A159TYR, A163THR, A166GLU, A167TRP, C1LEU, C5TYR, C8TYR, D26ASP, D29SER, D31SER, D50TYR, D68LYS, D93THR, D98THR, D99ASP, D100SER, D101TRP, E95ARG, E101GLY, E103PRO |
| 1BP3 | A21HIS, A25PHE, A160TYR, A164TYR, A167ARG, A168LYS, A171ASP, A172LYS, A174GLU, A176PHE, A178ARG, A179ILE, A180VAL |
| 1C4Z | A634VAL, A635LEU, A638SER, A639LEU, A641ASP, A642LEU, A653MET, A655ILE, A656THR, A659ILE, A660SER, A690PHE, D6ARG, D33LEU, D60GLU, D62PRO, D63PHE, D64LYS, D95TRP, D97PRO |
| 1E50 | A66ASP, A69ASN, A106MET, A109ASN, A113TYR, A114SER, A149THR, A159VAL, A161THR, B58VAL, B63ASN, B65SER, B104ASN |
| 1GUA | A37GLU, A39SER, B66GLN, B68THR, B69VAL |
| 1JTD | A99GLN, A102LEU, A104GLU, A105TYR, A107PRO, A110GLU, A111LYS, A129MET, A216VAL, A270MET, B50ASN, B52ASP, B53TRP, B73TYR, B74PHE, B112ASN, B113TYR, B152TRP, B167ASP, B191TYR, B206ASP, B229ILE, B230PHE, B248TYR, B268GLU, B269TRP, B286ARG, B304ASN |
| 1K8R | A37GLU, A38ASP, A39SER, A41ARG, A57ASP, B81GLN, B83ARG, B101LYS, B160ARG |
| 1MI5 | D30THR, D30AASP, D31TYR, D33HIS, D48HIS, D50LEU, D94LEU, D96GLY, D97GLY, D99SER, D100TYR, D102GLY, D103LYS, E30VAL, E31SER, E50GLN, E51ASN, E94SER, E95SER, E97GLY, E98GLN, E100TYR, E106GLN |
| 2AK4 | A65GLN, A69THR, A154GLU, A155GLN, A163LEU, C4PRO, C5LEU, C6PRO, C7GLN, C10LEU, D29THR, D31TYR, D50ASN, D93SER, D96TYR, D97ASN, E30ASN, E51SER, E98LEU |
| 2BNR | A63GLU, A64THR, A65ARG, A66LYS, A67VAL, A68LYS, A70HIS, A72GLN, A73THR, A74HIS, A76VAL |
| 2NYY | A915GLN, A917PHE, A918ASN, A919LEU, A920GLU, A953PHE, A954ASN, A1062ASP, A1063THR, A1064HIS |
| 2NZ9 | A917PHE, A918ASN, A919LEU, A920GLU, A953PHE, A954ASN, A1062ASP, A1063THR, A1064HIS |
| 3C60 | C58GLN, C61LEU, C62GLN, C63ASN, C66VAL, C69HIS, C73VAL, D4GLN, D5LYS, D7LYS, D9ASN |
| 3M62 | A40ASP, A44LEU, A96GLY, A97TYR, B45ILE, B47SER, B50VAL, B69VAL, B71MET |
| 3M63 | A40ASP, A44LEU, A48THR, A96GLY, A97TYR, A100VAL, A104ILE |
| 3NGB | H47TRP, H50TRP, H52LYS, H54GLY, H55GLY, H57VAL, H58ASN, H59TYR, H61ARG, H69MET, H100TYR, H100AASN, H100BTRP, L91TYR, L96GLU, L97PHE |
| 3QHY | B50ASN, B52ASP, B53TRP, B73TYR, B74PHE, B112ASN, B113TYR, B152TRP, B167ASP, B191TYR, B206ASP, B209PHE, B229ILE, B230PHE, B268GLU, B269TRP, B304ASN |
| 3QIB | B64GLN, B66GLU, B69GLU, B76ASP, B77THR, B81HIS, P7TYR, P9LYS, P12THR |
| 3S9D | A15LEU, A26LEU, A30LEU, A33ARG, A144ARG, A148MET, A149ARG, A152SER, A153LEU, B43TYR, B44THR, B45ILE, B46MET, B47SER, B76HIS, B77GLU, B80VAL, B98ASN, B100TRP, B103ILE |
| 3SE3 | A70TYR, A96PHE, A131LEU, A132ASP, A135SER, A238PHE, A241ARG |
| 3SE4(1) | A70TYR, A131LEU, A132ASP, A135SER, A181THR, A238PHE, A241ARG, A242ASN |
| 3SE4(2) | B28PRO, B28PRO, B32LEU, B32LEU, B148MET, B148MET, B152LYS, B152LYS, C44THR, C46MET, C47SER, C77GLU, C80VAL, C103ILE |
| 3SE8 | H30ARG, H47TRP, H50TRP, H52LYS, H53LEU, H54TRP, H55GLY, H57VAL, H58SER, H59TYR, H61ARG, H64GLN, H71ARG, H100CASP, H100DPHE, L50ASP, L91PHE, L96GLU, L97PHE |
| 3SE9(1) | H34LEU, H47TRP, H50TRP, H52BVAL, H53THR, H54GLY, H56VAL, H57ASN, H59GLY, H64ARG, H71ARG, H99THR, H100GLY, H100AGLY, H100CGLY, L31GLY, L91LEU, L96GLU, L97PHE |
| 3SZK | C125TYR, C126TYR, C127HIS, C129PHE |
| 4G0N | A21ILE, A37GLU, A38ASP, A39SER, B66GLN, B68THR, B69VAL, B88VAL |
| 4HFK | B63LEU, B64GLU, B66SER, B68LEU |
| 4HRN | D25LEU, D51SER, D52VAL, D55PHE |
| 4JFF | C1GLU, C4GLY, C6GLY, C7ILE, C8LEU, C9THR |
| 4OFY | A54GLN, A56MET, A60PHE, A105GLN, A107SER, A108GLU, D61LEU |
| 4OZG | H57GLN, H108PHE, H109ARG, H110PHE, H114ASP |
| 4P23 | C55ASP, C57GLN, C60LEU, C61GLN, C62ASN, C65VAL, C68HIS, C72VAL, D-16LYS, D-17ASN, D-19LYS, D-21LYS, D-22GLN, D-24GLU, D67ILE, D70ARG |
| 4P5T | C55ASP, C57GLN, C60LEU, C61GLN, C62ASN, C65VAL, D-17ASN, D-19LYS, D-21LYS, D-22GLN, D-24GLU, D77THR, D81HIS |
| 4RS1 | A99GLN, A109ASN, A112ASP, A115LEU |
| 5C6T | A280TYR, A283THR, A290PHE, A292GLU, A299ILE, H32SER, H102ASN, H103TYR, H104PHE, L50TYR, L51ARG |
| 5M2O | A75ASN, A77LYS, A121HIS, A124ASN, B39ILE, B40SER, B43VAL, B47GLN, B83GLN, B87LEU |
| 5XCO | B6PRO, B7LEU, B8TYR, B9ILE, B12ASP |
